# The Coordination of Centromere Replication, Spindle Formation, and Kinetochore–Microtubule Interaction in Budding Yeast

**DOI:** 10.1371/journal.pgen.1000262

**Published:** 2008-11-21

**Authors:** Hong Liu, Fengshan Liang, Fengzhi Jin, Yanchang Wang

**Affiliations:** 1 Department of Biological Sciences, Florida State University, Tallahassee, Florida, United States of America; 2 Department of Biomedical Sciences, College of Medicine, Florida State University, Tallahassee, Florida, United States of America; 3 Institute of Molecular Biophysics, Florida State University, Tallahassee, Florida, United States of America; National Institute of Diabetes and Digestive and Kidney Diseases, United States of America

## Abstract

The kinetochore is a protein complex that assembles on centromeric DNA to mediate chromosome–microtubule interaction. Most eukaryotic cells form the spindle and establish kinetochore–microtubule interaction during mitosis, but budding yeast cells finish these processes in S-phase. It has long been noticed that the S-phase spindle in budding yeast is shorter than that in metaphase, but the biological significance of this short S-phase spindle structure remains unclear. We addressed this issue by using *ask1-3*, a temperature-sensitive kinetochore mutant that exhibits partially elongated spindles at permissive temperature in the presence of hydroxyurea (HU), a DNA synthesis inhibitor. After exposure to and removal of HU, *ask1-3* cells show a delayed anaphase entry. This delay depends on the spindle checkpoint, which monitors kinetochore–microtubule interaction defects. Overproduction of microtubule-associated protein Ase1 or Cin8 also induces spindle elongation in HU-arrested cells. The spindle checkpoint-dependent anaphase entry delay is also observed after *ASE1* or *CIN8* overexpression in HU-arrested cells. Therefore, the shorter spindle in S-phase cells is likely to facilitate proper chromosome–microtubule interaction.

## Author Summary

The interaction of spindle microtubules with chromosomes is essential for proper segregation of chromosomes into two daughter cells. Most eukaryotic cells form spindles and establish the chromosome–microtubule interaction after DNA replication, but the chromosomes in budding yeast are attached by spindle microtubules before the completion of DNA replication. Interestingly, when yeast cells are arrested in S-phase by hydroxyurea, a DNA synthesis inhibitor, they show a unique spindle structure that is shorter than the metaphase spindle. The focus of this study is to address how the shorter S-phase spindle benefits chromosome–microtubule interaction. We found that the increase in spindle length in yeast cells that are arrested in S-phase results in an improper chromosome–microtubule interaction. It is likely that the shorter S-phase spindle facilitates chromosome capture by microtubules as well as the generation of tension on chromosomes, which is critical for flawless chromosome segregation.

## Introduction

One of the most important events during the cell cycle is chromosome segregation. This process requires each duplicated chromosome to be captured by microtubules emanating from opposite spindle poles in order to establish bipolar attachment. The establishment of bipolar attachment satisfies the spindle checkpoint, leading to the degradation of anaphase inhibitor Pds1 by APC (anaphase-promoting complex) and the subsequent cleavage of cohesins [1],[2],[3]. The presence of a single unattached kinetochore is sufficient to activate the spindle checkpoint and thereby to prevent anaphase entry [4]. Improperly attached chromosomes lead to tension defects, which activate the tension checkpoint and prevent anaphase entry as well [5]. Unlike other higher eukaryotic organisms, in which kinetochore-microtubule interaction is established during M-phase, the budding yeast undergoes spindle formation and chromosome capture during S-phase [6]. The real time analysis of spindle in budding yeast reveals several stages during cell cycle [7]. Following spindle pole body duplication, the spindle appears as a short bar on the side of the nucleus, <1.2 µM in length. After further spindle poles separation, a bipolar spindle (1.5–2 µM) bisects the nucleus. On the basis of bud size, these two stages may represent S- and M-phase spindles.

In budding yeast, chromosomes are attached to microtubules during most of the cell cycle, but the duplication of centromeric DNA results in the dissociation of kinetochore proteins from centromeres, which is expected to disrupt kinetochore-microtubule interaction [8]. Centromere replication has been shown to result in the dissociation of chromosomes from microtubules for 1–2 min. The detached chromosomes are recaptured by microtubules immediately after completion of centromere replication [8]. Previous work shows that newly synthesized Cse4, the histone-H3 variant specific for centromeres in budding yeast, replaces old protein during DNA replication. Once assembled, Cse4-GFP is a stable component of centromeres during mitosis [9]. Together, the observations support the conclusion that the kinetochore-microtubule interaction is disrupted and reestablished during S-phase in budding yeast.

Hydroxyurea (HU) slows down DNA synthesis by depleting dNTP pool, and the presence of high concentrations of HU arrests yeast cells in S-phase with a short spindle. Mutant cells deficient in S-phase checkpoint exhibit elongated spindle structure in HU-arrested cells [10],[11], but the cause for spindle elongation in S-phase checkpoint mutants remains uncertain. Results from the Bachant lab support the notion that the failure of bipolar attachment results from the incomplete centromere replication contributes to spindle elongation in S-phase checkpoint mutants in the presence of HU [10]. In support of this notion, they observed that some kinetochore mutants, *ask1-3*, *mif2-2*, and *ipl1-321*, failed to maintain short spindles in the presence of HU when incubated at the nonpermissive temperature. As these mutants exhibit defects in the establishment of chromosome bipolar attachment [12],[13],[14], the bipolar attachment is believed to restrain spindle elongation during S-phase. Data from another lab suggest that the down-regulation of microtubule-associated proteins, Cin8 and Stu2, by S-phase checkpoint is the key to the short spindle structure in HU-arrested cells [11]. Deletion of Cin8, a protein required for spindle elongation, has been shown to suppress the spindle elongation phenotype in S-phase checkpoint mutants. Therefore, both bipolar attachment and down-regulation of proteins responsible for spindle elongation are probably required to prevent spindle elongation in HU-arrested yeast cells. A key question that remains open is why yeast cells keep a short spindle structure during S-phase. One way to address this issue is to determine what happens to yeast cells when spindles elongate during S-phase.

We found that *ask1-3* mutant cells exhibited partially elongated spindles when arrested with HU at room temperature, even though the mutant cells have no noticeable cell cycle defects in the absence of HU treatment. We treated *ask1-3* mutant cells with HU and followed cell cycle progression after HU was washed off. Strikingly, the mutant cells exhibited normal DNA synthesis, but showed a dramatic anaphase entry delay that depends on the spindle checkpoint, indicating improper kinetochore-microtubule interactions. Deletion of *CIN8* suppressed the premature spindle elongation as well as the anaphase entry delay in *ask1-3* mutants after HU treatment. To further confirm that the premature spindle elongation contributes to the anaphase entry delay, we enforced spindle elongation in S-phase cells by overexpressing *ASE1* or *CIN8*, because their overexpression has been reported to induce spindle elongation in HU-arrested cells [11],[15]. We also found that Ase1- or Cin8-induced spindle elongation in HU-arrested cells also led to spindle checkpoint-dependent anaphase entry delay after HU was washed away. Therefore, we conclude that the short S-phase spindle is required for proper kinetochore-microtubule interaction in budding yeast.

## Results

### The Examination of Kinetochore Integrity in HU-Arrested Cells

In budding yeast, kinetochores interact with microtubules during most of the cell cycle, but a transient chromosome dissociation from microtubules occurs during S-phase [8]. Previous evidence indicates that centromeres are duplicated in early S-phase, because they are close to early firing origins [16],[17],[18]. Hydroxyurea (HU) slows down DNA synthesis, but it remains untested whether HU treatment delays kinetochore assembly. For this purpose, we examined the association of kinetochore proteins with centromeric DNA in HU-arrested yeast cells by chromatin immunoprecipitation (ChIP) assay. Ask1 is one of the subunits of the DASH complex, which encircles microtubules and mediates a stable kinetochore-microtubule interaction [19],[20],[21]. The kinetochore-microtubule interaction can therefore be reflected by the association of Ask1 protein with centromeric DNA. Nnf1 is a component of the Mtw1 kinetochore complex, which is required to bridge the DASH-centromere interaction [20]. The association of Nnf1 with centromeric DNA would indicate the assembly of core kinetochore structure.

**Figure 1:**
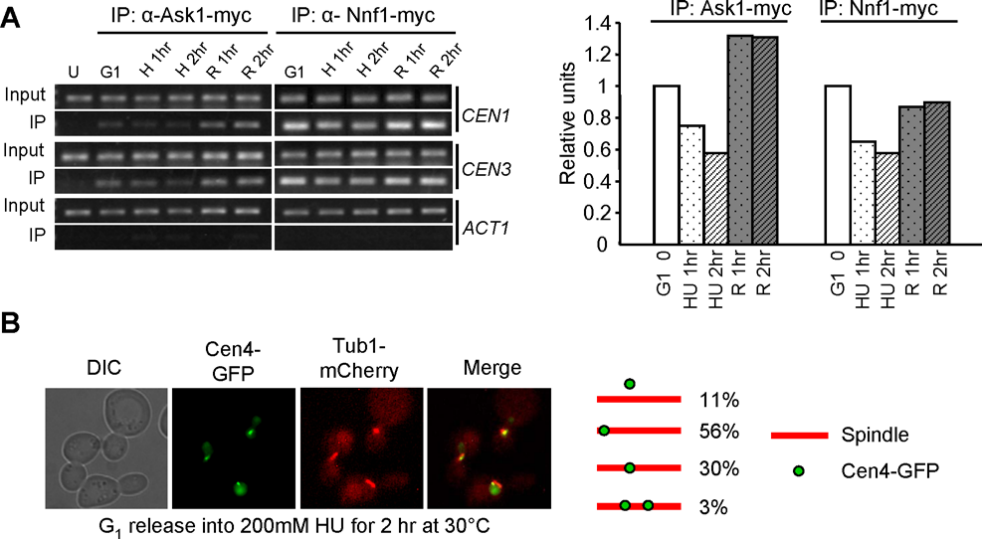
Kinetochore-microtubule interaction in HU-treated cells. (A) Examination of kinetochore integrity and kinetochore-microtubule interaction during S-phase. G_1_-arrested cells with untagged (U) or myc-tagged *ASK1* and *NNF1* were released into YPD medium containing 200 mM HU for 2 hrs at 30°C and then released into 30°C YPD medium. Cells were collected at indicated time points for ChIP assay. DNA from total (input) and immunoprecipitates (IP) were used for PCR reaction with primers specific to *CEN1*, *CEN3*, and *ACT1*. H and R stand for HU and Release, respectively. The quantified association of Ask1 and Nnf1 with *CEN3* is shown on the right panel. (B) Centromere localization in HU-arrested yeast cells. G_1_-arrested *TUB1-mCherry CEN4-GFP* cells were released into YPD medium containing 200 mM HU. After incubation at 30°C for 2 hrs, cells were collected for the examination of fluorescence signals. The percentage of cells with different Cen4-GFP distribution is shown on the right.

So that the association of Ask1 and Nnf1 with centromeric DNA could be examined, cells with myc-tagged Ask1 or Nnf1 were synchronized in G_1_ phase and then released into HU medium. Consistent with those of the previous studies, our results demonstrate the association of Ask1 and Nnf1 with centromeric DNA in cells arrested in G_1_ phase [12], indicating kinetochore-microtubule interaction at this cell cycle stage. Interestingly, the association decreased after release into HU medium for 1 and 2 hrs, suggesting the failure of kinetochore assembly on some centromeres (Figure 1A). We speculate that either unduplicated centromeres or closely localized replication machinery could prevent kinetochore assembly in HU-arrested cells. After HU wash-off, increased centromere binding of Ask1 and Nnf1 was observed, indicating kinetochore reassembly and the establishment of kinetochore-microtubule interaction (Figure 1A). These observations provide direct evidence for the presence of some centromeres that lack the association of kinetochore proteins in HU-arrested yeast cells.

We also examined the localization of GFP-marked centromere of chromosome IV (Cen4-GFP) and mCherry-tagged Tub1 [22],[23]. The majority of HU-arrested cells exhibited a single GFP dot positioned either close to the middle of the spindle (30%) or near to one of the spindle ends (56%). The chromosome IV in cells with a Cen4-GFP dot near to one spindle end is likely to be monopolar attached, because in a certain fraction of cells, centromeres are not yet replicated in HU and only monolopar attachment can form under these circumstances. We also found that some Cen4-GFP dots were away from the spindle axis (11%), and we reason that these chromosomes are either detached or monopolar attached. After bipolar attachment, the applied tension on chromosomes results in a transient sister centromere separation and two dots can be visualized with GFP-marked centromeres [24],[25]. About 3% of cells exhibited two separated Cen4-GFP dots in the presence of HU for 2 hr (Figure 1B). It has been shown that 29% HU-treated cells exhibit two Cen15-GFP dots, presumably due to the chromosome specificity or different experimental conditions [24]. Taken together, we conclude that detached, monopolar attached, or bipolar attached chromosomes are all present in HU-arrested cells. As we observed decreased association of kinetochore proteins with centromeres in HU-treated cells, it is likely that only some chromosomes are competent to establish bipolar attachment in the presence of HU.

### *ask1-3* Mutant Cells Show Delayed Anaphase Entry after HU Treatment

**Figure 2:**
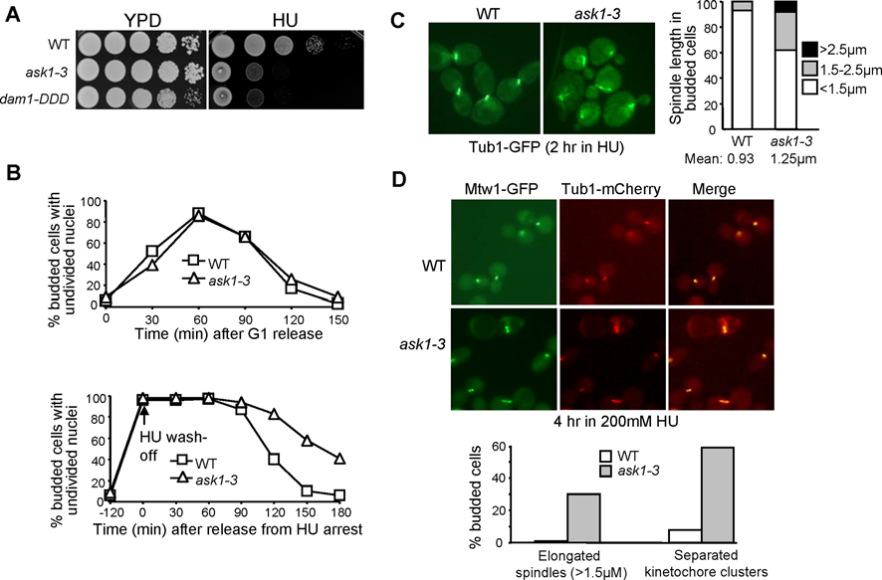
Premature spindle elongation and abnormal kinetochore distribution in HU-arrested *ask1-3* mutant cells. (A) *ask1-3* and *dam1-DDD* mutants show HU sensitivity. Cells with the indicated genotypes were grown to saturation. After 10-fold dilution, the cells were spotted onto YPD plates with and without 100 mM HU. The plates were incubated at 25°C for 4 days before they were scanned. (B) HU treatment results in dramatic anaphase entry delay in *ask1-3* cells. G_1_-arrested WT and *ask1-3* cells were released into YPD medium with and without 200 mM HU at 25°C. HU was washed off after 2 hr incubation and the cells were then resuspended in 25°C YPD medium. α-factor was added to block the second round of cell cycle. Cells were collected at the indicated time points for DAPI staining. (C) The spindle length in WT and *ask1-3* mutants after HU treatment. G_1_-arrested cells (*TUB1-GFP* and *ask1-3 TUB1-GFP*) were released into 25°C YPD medium containing 200 mM of HU for 2 hrs. The cells were then collected, fixed with formaldehyde, and subjected to fluorescence microscopy. The spindle morphology is shown in the left panel and the percentage of cells with different spindle lengths is shown on the right. More than 100 cells were measured for spindle length for each strain. (D) Premature spindle elongation and abnormal kinetochore localization in HU-arrested *ask1-3* cells. G_1_-arrested *TUB1-mCherry MTW1-3GFP* and *ask1-3 TUB1-mCherry MTW1-3GFP* were released into medium containing 200 mM HU and incubated for 4 hrs at 25°C. Cells were collected and fixed for fluorescence microscopy. Mtw1-GFP localization and spindle morphology in the representative cells are shown in the top panel; the percentage of cells with elongated spindles or two separated kinetochore clusters is shown in the bottom panel.

*ask1-1* was identified from a genetic screen for mutants that exhibited elongated spindles in the presence of HU, but further investigations have demonstrated that Ask1 is a kinetochore component [12]. We generated a temperature-sensitive mutant, *ask1-3*, that showed elongated spindles and missegregated chromosomes when incubated at the nonpermissive temperature [12]. *ask1-3* mutants also exhibited elongated spindles in HU-arrested cells when incubated at 37°C [10]. We determined whether this mutant allele also shows HU sensitivity by growing the mutant cells on HU plates. Interestingly, *ask1-3* mutants failed to form colonies on HU plates even when grown at 25°C (Figure 2A). We also examined the HU sensitivity of another DASH mutant, *dam1-DDD*, in which the Ipl1/Aurora-B phosphorylation sites are mutated to aspartic acids to mimic the constitutive phosphorylation status [26]. *dam1-DDD* mutants also exhibited HU sensitivity (Figure 2A), suggesting that compromised DASH function results in HU sensitivity.

To determine the nature of the HU sensitivity, we examined the viability of *ask1-3* cells after incubation in the presence of HU. No obvious viability loss was observed for *ask1-3* mutant after a 5-hr treatment with HU, suggesting that the HU sensitivity is unlikely to be a result of defective checkpoints. Comparison of the cell cycle progression in wild-type (WT) and *ask1-3* mutant cells with and without HU exposure revealed that, in the absence of HU, they showed similar cell cycle progression when incubated at 25°C as indicated by the nuclear division kinetics. However, *ask1-3* cells exhibited dramatically delayed nuclear division after release from 2-hr HU treatment (Figure 2B), suggesting that some defects halt anaphase entry in *ask1-3* cells after HU treatment. We reason that the defects in *ask1-3* mutants are insufficient to slow down cell cycle when grown at 25°C without disturbance, but after HU treatment, the consequence of compromised Ask1 function blocks anaphase entry.

### *ask1-3* Mutants Exhibit Elongated Spindles and Abnormal Kinetochore Localization in the Presence of HU

In the presence of HU, WT cells arrest with a 1–2 µM spindle [10]. *ask1-1* mutant cells have been shown to exhibit abnormal spindle structure in the presence of HU [12]. In addition, the spindles became elongated in *ask1-3* mutant cells when incubated in 37°C medium containing HU [10]. Since *ask1-3* mutants show HU sensitivity when incubated at room temperature, we examined spindle morphology in HU-arrested *ask1-3* mutant cells incubated at 25°C and found that some cells showed partially elongated spindles (Figure 2C). Only 7% of WT cells had a spindle longer than 1.5 µM, but the number increased to 38% for *ask1-3* mutants. We further determined whether the elongated spindle leads to abnormal kinetochore distribution. For this purpose, WT and *ask1-3* mutant cells with mCherry-tagged Tub1 and GFP-tagged Mtw1, a kinetochore protein, were incubated in HU medium at 25°C for 4 hrs [23],[27]. About 30% *ask1-3* mutant cells exhibited spindle length longer than 1.5 µM. Interestingly, 60% of *ask1-3* cells showed two clearly separated kinetochore clusters (Mtw1-GFP), whereas the majority of WT cells exhibited one unseparated kinetochore cluster (Figure 2D). *dam1-DDD* mutant cells also exhibited partially elongated spindles and two separated kinetochore clusters in the presence of HU (data not shown), indicating that a functional DASH complex is required to restrain spindle elongation during S-phase.

**Figure 3:**
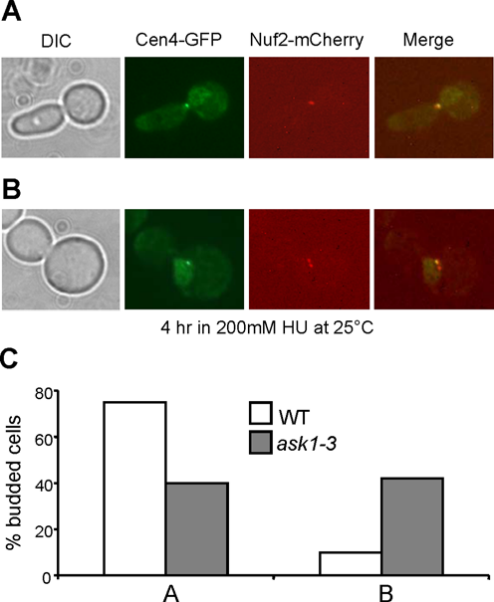
The abnormal kinetochore localization in HU-arrested *ask1-3* cells is not a result of bipolar attachment. G_1_-arrested *NUF2-mCherry CEN4-GFP* and *ask1-3 NUF2-mCherry CEN4-GFP* were released into YPD medium containing 200 mM HU for 4 hrs at 25°C. Cells were collected and fixed for fluorescence microscopy. The localization of Nuf2-mCherry and Cen4-GFP falls into two categories (A and B). (A) A representative cell with a single Nuf2-mCherry cluster and Cen4-GFP dot. (B) A representative cell with two Nuf2 clusters but one Cen4-GFP dot. (C) The percentage of cells with phenotypes shown in A and B.

To understand the nature of the two separated kinetochore clusters in HU-treated *ask1-3* mutant cells, we examined the localization of Cen4-GFP and mCherry-tagged kinetochore protein Nuf2 [22],[28]. In agreement with the result shown in Figure 2, 60% of *ask1-3* cells exhibited two Nuf2-mCherry clusters after incubation in HU medium for 4 hrs at 25°C, whereas only 10% of WT cells showed two separated Nuf2 foci (Figure 3). In the *ask1-3* cells with two Nuf2 clusters, only one single Cen4-GFP dot was observed, which colocalizes with one of the Nuf2-mCherry clusters. This result suggests that the appearance of two kinetochore clusters in HU-arrested *ask1-3* cells is likely a result of random movement of chromosomes close to one of the spindle poles.

### *ask1-3* Cells Exhibit Spindle Checkpoint-Dependent Anaphase Entry Delay

**Figure 4:**
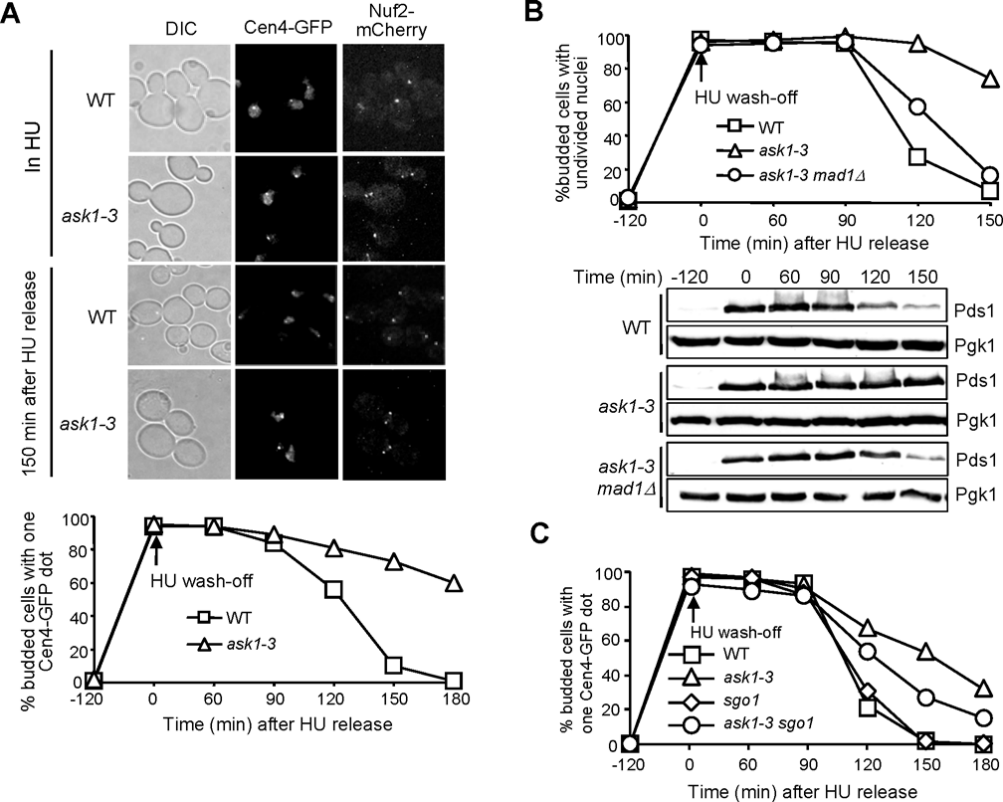
The anaphase entry delay in *ask1-3* mutant cells after HU treatment. (A) *ask1-3* cells exhibit delayed chromosome segregation after HU treatment. G_1_-arrested *NUF2-mCherry CEN4-GFP* and *ask1-3 NUF2-mCherry CEN4-GFP* cells were released into YPD medium containing 200 mM HU and incubated at 25°C. Two hours later, HU was washed off and the cells were released into YPD medium. α-factor was added back to block the second round of cell cycle. Cells were collected for the examination of fluorescence signals. The localization of Nuf2 and Cen4 at the indicated times is shown in the top panel. The percentage of budded cells with one Cen4-GFP dot is shown in the bottom panel. (B) *ask1-3* cells exhibit spindle checkpoint-dependent anaphase entry delay after HU treatment. G_1_-arrested *PDS1-myc*, *ask1-3 PDS1-myc*, and *ask1-3 mad1Δ PDS1-myc* cells were released into YPD medium containing 200 mM HU for 2 hrs. After HU was washed off, the cells were released into YPD medium and incubated at 25°C. The cells were collected at the indicated time points for protein preparation and DAPI staining. Pgk1 protein levels are shown as a loading control. (C) Deletion of tension checkpoint *SGO1* partially suppresses the anaphase entry delay in *ask1-3* mutants after HU treatment. Cells with indicated genotypes were treated as described in B. Here shows the percentage of large budded cells with a single Cen4-GFP dot after HU wash off.

We observed partially elongated spindles and abnormal kinetochore distribution in HU-treated *ask1-3* mutants. We next asked why *ask1-3* mutants show HU sensitivity by comparing cell cycle progression in WT and *ask1-3* mutant cells after HU exposure. For this purpose, G_1_-arrested cells with Nuf2-mCherry and Cen4-GFP were released into 200 mM HU for 2 hrs at 25°C. The fluorescence signals were examined after HU was washed off. As shown in Figure 4A, both WT and *ask1-3* mutant cells showed a single GFP dot before release from HU arrest. 150 min after release from HU arrest, most of WT cells showed two GFP dots, one in each daughter cell, indicating anaphase entry. In contrast, 70% of *ask1-3* cells exhibited two kinetochore clusters with one Cen4-GFP dot (Figure 4A). To exclude the possibility that unfinished DNA replication in *ask1-3* mutants prevents sister chromatid separation, we used FACS analysis to monitor DNA synthesis and found that both WT and *ask1-3* strains finished DNA synthesis after release from HU arrest for 60 min (data not shown), suggesting that the delayed sister separation is not a result of DNA synthesis defects. The appearance of the single Cen4-GFP dot in *ask1-3* mutants after release from HU arrest for a long time could be a result of either monopolar attachment or the lack of tension, as tension generation results in transient sister centromere separation before anaphase entry [24],[25].

Chromosome-microtubule interaction defects have been shown to activate the spindle checkpoint, which stabilizes Pds1 protein to prevent anaphase entry [29]. To examine the kinetochore-microtubule interaction defects in HU-treated *ask1-3* mutants, we compared Pds1 protein levels and nuclear division kinetics in *ask1-3* mutant cells after HU treatment in the presence and absence of the spindle checkpoint. As shown in Figure 4B, WT cells accumulated high levels of Pds1 protein in the presence of HU, but Pds1 protein levels decreased dramatically after release from HU arrest for 120 min. In contrast, Pds1 protein levels remained persistent in *ask1-3* cells, consistent with the delayed nuclear division. However, deletion of *MAD1*, which encodes one of the spindle checkpoint components, eliminated the delay in both Pds1 degradation and nuclear division in *ask1-3* cells (Figure 4B). The results suggest that *ask1-3* mutant cells exhibit kinetochore-microtubule interaction defects after HU treatment, which activate the spindle checkpoint to prevent anaphase entry.

To clarify whether the appearance of a single Cen4-GFP dot in *ask1-3* mutants after HU treatment is due to the failure of bipolar attachment, we followed Cen4-GFP separation and nuclear division in *ask1-3* and *ask1-3 mad1Δ* mutants after they were exposed to HU for 2 hr. After release from HU arrest for 120 min, the cells were collected for DAPI staining. Surprisingly, only 4% of *ask1-3 mad1Δ* cells exhibited Cen4-GFP missegregation, i.e., Cen4-GFP stays with one of the divided nuclei, whereas no missegregation was observed in *mad1Δ* and *ask1-3* mutants. As a result, 40% of *ask1-3 mad1Δ* cells lost viability after treatment with 200 mM HU for 2 hr.

As most of *ask1-3 mad1Δ* mutant cells are able to separate Cen4-GFP after HU treatment, defects other than the failure of bipolar attachment may also contribute to the appearance of a single Cen4-GFP dot in *ask1-3* mutants. It is possible that most chromosomes are able to establish bipolar attachment, but the failure of tension generation prevents the transient separation of sister centromeres before anaphase. To test this possibility, we visualized Cen4-GFP separation in *ask1-3* and *ask1-3 sgo1Δ* mutants after HU treatment. It was obvious that the deletion of the tension checkpoint *SGO1* suppressed the sister centromere separation delay in *ask1-3* mutants [30], although the suppression was not as complete as *mad1Δ* (Figure 4C). The results suggest that defects in both chromosome attachment and tension generation are responsible for the anaphase entry delay in *ask1-3* mutants after HU treatment.

### Deletion of *CIN8* Partially Suppresses the HU Sensitivity of *ask1-3* Mutants

**Figure 5:**
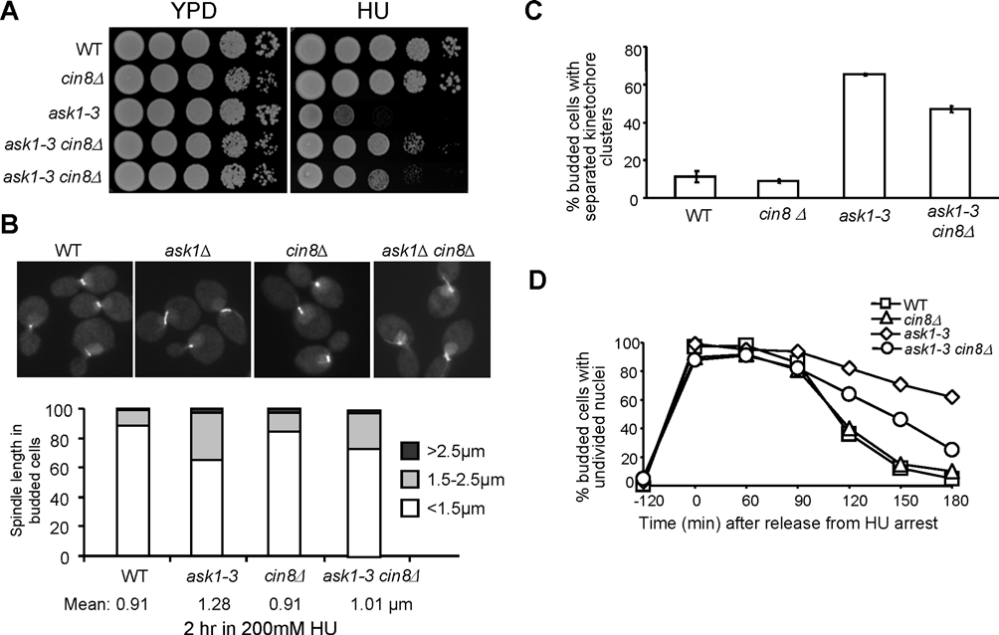
Deletion of *CIN8* partially suppresses the phenotypes of *ask1-3* mutants. (A) Deletion of *CIN8* partially suppresses the HU sensitivity of *ask1-3* mutants. Cells with indicated genotypes were grown to saturation and then 10-fold diluted and spotted onto YPD plates with and without 100 mM HU. The plates were incubated at 25°C for 4 days before being scanned. (B) *cin8Δ* mutation suppresses spindle elongation in HU-treated *ask1-3* mutants. Asynchronous cells with indicated genotypes were grown to mid-log phase and HU was then added to the cell cultures to a final concentration of 200 mM. After 2 hr incubation at 25°C, cells were fixed for fluorescence microscopy. The spindle morphology in some representative cells is shown in the top panel and the percentage of cells with different spindle length and the average spindle length are shown in the bottom panel. (C) *cin8Δ* mutation partially suppresses the abnormal kinetochore distribution phenotype in HU-treated *ask1-3* mutant cells. G_1_-arrested WT, *ask1-3* and *cin8Δ ask1-3* cells with *MTW1-GFP* were released into YPD medium containing 200 mM HU and incubated at 25°C for 4 hrs. Cells were collected and fixed for the examination of Mtw1-GFP signal. Shown here is the percentage of cells with two clear GFP clusters. (D) Deletion of *CIN8* partially suppresses the anaphase entry delay in *ask1-3* mutants after HU treatment. G_1_-arrested WT, *cin8Δ*, *ask1-3* and *cin8Δ ask1-3* cells were released into YPD medium containing 200 mM HU for 2 hrs. After HU was washed off, the cells were released into 25°C YPD medium and collected at the indicated time points for DAPI staining. The percentage of budded cells with undivided nuclei is shown.

*ask1-3* mutant cells show prematurely elongated spindles in the presence of HU, which could contribute to the observed HU sensitivity. If that is the case, prevention of spindle elongation would suppress the HU sensitivity. As Cin8 has been shown to be involved in spindle elongation during mitosis [31], we compared the HU sensitivity of *ask1-3* and *ask1-3 cin8Δ* mutants. In agreement with our prediction, *ask1-3 cin8Δ* grew much better than *ask1-3* single mutant cells on HU plates, although strains differed in growth (Figure 5A). Ase1 is a spindle midzone protein required for spindle elongation and stabilization [32]. Deletion of *ASE1* also suppressed the HU sensitivity of *ask1-3* mutants, but the suppression was less significant compared to *cin8Δ* (data not shown).

To address why *cin8Δ* deletion suppresses the HU sensitivity of *ask1-3*, we first examined the spindle morphology in *ask1-3* and *ask1-3 cin8Δ* cells after HU treatment for 2 hrs. Strikingly, the average spindle length decreased from 1.28 µM in *ask1-3* to 1.01 µM in *ask1-3 cin8Δ* mutants after incubation in HU (Figure 5B). The number of cells with spindle length longer than 1.5 µM also decreased in the double mutants. The examination of the kinetochore cluster separation in *ask1-3 cin8Δ* mutants in the presence of HU indicated that the phenotype in *ask1-3* mutants was partially suppressed by the deletion of *CIN8* (Figure 5C). We then compared the cell cycle progressions of the single- and double-mutant cells after HU treatment. The delayed nuclear division in *ask1-3* mutants was suppressed significantly in *ask1-3 cin8Δ* double mutants, albeit not completely (Figure 5D). Therefore, the premature spindle elongation in HU-arrested *ask1-3* mutant cells is likely responsible for the improper kinetochore-microtubule interaction, which contributes to the HU sensitivity.

### Ase1 or Cin8-Induced Spindle Elongation in HU-Arrested Cells also Causes Improper Kinetochore–Microtubule Interaction

**Figure 6:**
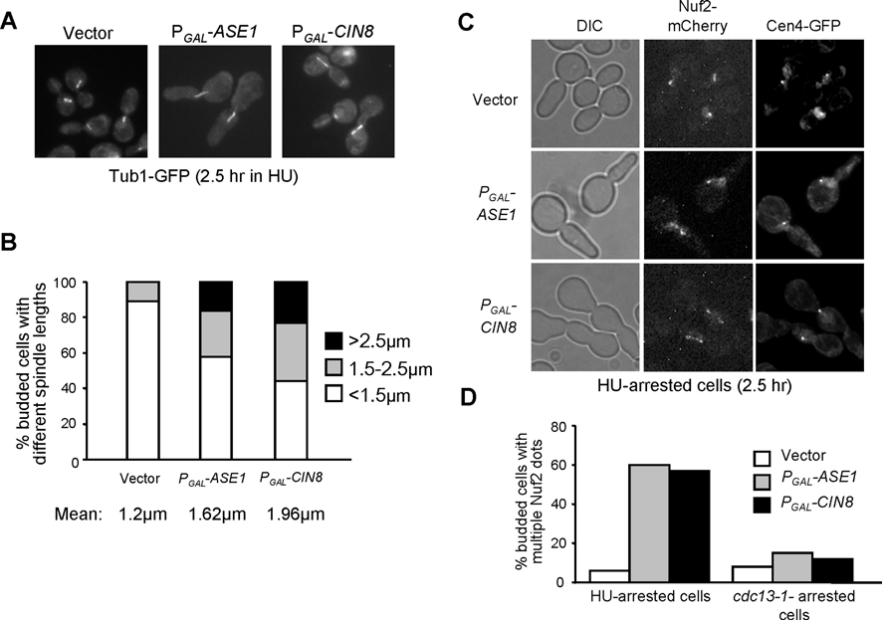
Overexpression of *ASE1* or *CIN8* results in spindle elongation and abnormal kinetochore distribution in the presence of HU. (A) Overexpression of *ASE1* or *CIN8* leads to spindle elongation in HU-arrested cells. G_1_-arrested *TUB1-GFP* cells with a vector, *P_GAL_-ASE1* or *P_GAL_-CIN8* plasmid were released into galactose medium containing 200 mM HU and incubated at 30°C for 2.5 hrs. The cells were then collected and fixed for fluorescence microscopy. The spindle morphology in some representative cells is shown. (B) The percentage of cells with different spindle lengths (<1.5 µM; 1.5–2.5 µM; >2.5 µM). The spindle length of more than 100 cells was measured for each sample. (C) Overexpression of *ASE1* and *CIN8* results in scattered kinetochore distribution along the spindle in HU-arrested cells. *NUF2-mCherry CEN4-GFP* cells with a vector, *P_GAL_-ASE1*, or *P_GAL_-CIN8* plasmid were grown to mid-log phase in raffinose medium. Cells were synchronized in G_1_ phase with α factor and then released into galactose medium containing 200 HU and incubated at 30°C. Cells were collected after 4 hr incubation for the examination of fluorescence signals. (D) Overexpression of *ASE1* or *CIN8* induces abnormal kinetochore distribution in HU- but not in *cdc13-1*-arrested cells. G_1_-arrested *cdc13-1 NUF2-mCherry* cells with a vector or *P_GAL_-ASE1*, *P_GAL_-CIN8* plasmid were released into galactose medium and incubated at 34°C. Nuf2 distribution was examined after 4 hr incubation. Shown here is the percentage of budded cells with scattered Nuf2 signals in HU-arrested cells (experiment C) and in *cdc13-1*-arrested cells.

The results we obtained using *ask1-3* mutant support the conclusion that premature spindle elongation during S-phase leads to improper kinetochore-microtubule interaction, but we could not rule out the possibility that the compromised kinetochore function in *ask1-3* mutants directly contributes to the chromosome attachment defects during the recovery from HU arrest. Clarification of this issue will require alternative ways to induce spindle elongation without disturbing kinetochore function. Overexpression of *ASE1* has been shown to induce premature spindle elongation in HU-arrested cells [15]. Because there is no evidence indicating a direct kinetochore function of Ase1, a spindle midzone binding protein, the kinetochores should function normally in cells overexpressing *ASE1* [15],[32],[33]. We first confirmed Ase1-induced spindle elongation phenotype and found that 42% of cells overexpressing *ASE1* showed a spindle longer than 1.5 µM after incubation in HU for 2.5 hr, compared to 11% for the control cells (Figure 6A and B).

We have shown that the premature spindle elongation in HU-treated *ask1-3* mutants leads to abnormal kinetochore distribution. To determine whether this is the case in cells overexpressing *ASE1*, we synchronized cells harboring a vector or a *P_GAL_-ASE1* plasmid in G_1_ phase and released them into galactose medium containing 200 mM HU. After incubation for 4 hrs, *ASE1* overexpression resulted in scattered Nuf2-mCherry signals along the spindle in 61% of cells and all these cells exhibited a single Cen4-GFP dot (Figure 6C and D). This result indicates that the spindle elongation enforced by *ASE1* overexpression also causes abnormal kinetochore distribution. It has been shown that overexpression of *CIN8* leads to spindle elongation in HU-arrested cells [11]. Next, we examined spindle elongation and kinetochore distribution in cells overproducing Cin8 and found that these cells showed a more dramatic phenotype than those overexpressing *ASE1* (Figure 6). Cdc13 is the telomere protein and telomere ends are not protected in *cdc13-1* mutants; the DNA damage checkpoint is therefore activated and arrests cells at preanaphase [34]. To determine whether this phenotype is specific to cells in S-phase, we also overexpressed *ASE1* and *CIN8* in *cdc13-1*-arrested cells. In contrast to HU-arrested cells, no obvious abnormal kinetochore distribution was observed in *cdc13-1*-arrested cells overexpressing *ASE1* or *CIN8* (Figure 6D), suggesting that only S-phase cells are sensitive to enforced spindle elongation.

**Figure 7:**
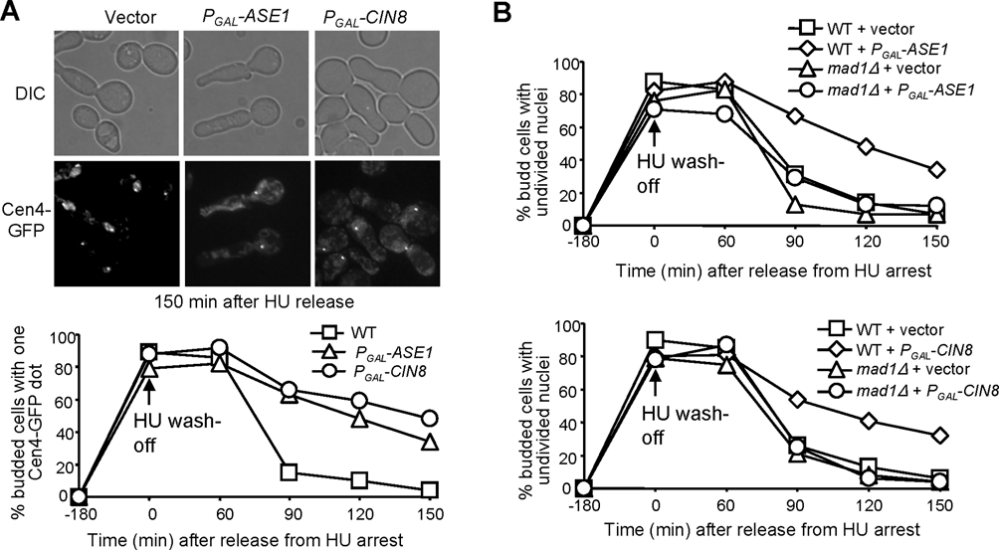
Overexpression of *ASE1* or *CIN8* in HU-arrested cells causes anaphase entry delay. (A) Overexpression of *ASE1* or *CIN8* in HU-arrested cells causes delayed anaphase entry after HU is washed off. G_1_-arrested *CEN4-GFP* cells with a vector, *P_GAL_-ASE1* or *P_GAL_-CIN8* plasmid were released into galactose medium containing 200 mM HU for 3 hrs at 30°C. HU was washed off and the cells were released into glucose medium and incubated at 30°C. α-factor was added to block the second round of cell cycle. Cells were collected at the indicated times and fixed for fluorescence microscopy. The top panel shows cells collected at 2.5 hr after HU release. The percentage of budded cells with a single Cen4-GFP is shown in the bottom panel. (B) Overexpression of *ASE1* and *CIN8* leads to spindle checkpoint-dependent anaphase entry delay. G_1_-synchronized WT and *mad1Δ* cells with a vector, *P_GAL_-ASE1* or *P_GAL_-CIN8* plasmid, were released into galactose medium containing 200 mM HU for 3 hrs at 30°C. The cells were released into 30°C glucose medium containing α-factor. Cells were collected at the indicated times for DAPI staining. The percentage of budded cells with undivided nuclei is shown.

*ask1-3* mutants show delayed anaphase entry after HU treatment and we speculate that the partially elongated spindle is responsible for this delay. If that is the case, Ase1 and Cin8-induced spindle elongation in HU-arrested cells should also lead to anaphase entry delay. Therefore, we synchronized cells with either plasmid *P_GAL_-ASE1*, *P_GAL_-CIN8* or a vector in G_1_-phase and then released them into galactose medium containing 200 mM HU to induce Ase1 and Cin8 expression. After 3 hrs of incubation, HU was washed off and cells were resuspended in glucose medium at 30°C to terminate the expression of *ASE1* and *CIN8*. In the presence of HU, all cells exhibited one single Cen4-GFP dot, but 90 min after release from HU arrest, the majority of cells with a vector had two GFP dots, indicating the onset of anaphase. However, more than 60% of cells harboring *ASE1* or *CIN8* plasmid still exhibited a single Cen4-GFP dot (Figure 7A), indicating a delayed anaphase entry. To test the dependency of this delay on the spindle checkpoint, we repeated the HU wash-off experiment with WT and *mad1Δ* mutant cells. The nuclear division was followed after HU treatment by DAPI staining. Deletion of *MAD1* clearly suppressed the anaphase entry delay induced by the overexpression of *ASE1* or *CIN8* (Figure 7B). The Mad1-dependent anaphase entry delay suggests an improper kinetochore-microtubule interaction in HU-arrested cells overexpressing *ASE1* or *CIN8*.

The premature anaphase entry in *mad1Δ* mutant cells after Ase1 or Cin8 induction in the presence of HU may result in chromosome missegregation. To test this possibility, G1-arrested *CEN4-GFP* and *mad1Δ CEN4-GFP* cells with a vector, *P_GAL_-ASE1*, or *P_GAL_-CIN8* plasmid were released into 30°C galactose medium containing 200 mM HU for 3 hrs to induce Ase1 and Cin8 expression. Then HU was washed off and the cells were released into glucose medium and incubated at 30°C. After 100 min, cells were stained with DAPI to examine nuclear division and Cen4-GFP separation. We found that 5% *mad1Δ* cells with *P_GAL_-ASE1* showed chromosome missegregation, whereas *CIN8* overexpression resulted in chromosome missegregation in 18% of *mad1Δ* mutant cells. No chromosome missegregation was observed in the control cells. One explanation for the difference between Ase1 and Cin8 is that overexpression of Cin8, but not Ase1, may disrupt kinetochore function, because Cin8 has been shown to bind to kinetochores [35],[36]. Together, we conclude that premature spindle elongation in HU-arrested cells induced by overexpression of *ASE1* or *CIN8* also leads to improper chromosome-microtubule interaction, which activates the spindle checkpoint to block anaphase entry.

## Discussion

The budding yeast exhibit a short spindle structure when DNA synthesis is blocked with HU [10]. Although previous work demonstrates that the S-phase checkpoint is essential to keep the spindle short in HU-arrested cells [37], the biological significance of the short spindle remains unknown. The characterization of the cell cycle defects resulted from elongated spindles in S-phase-arrested cells enables us to clarify this issue. We found that *ask1-3* mutant cells exhibited partially elongated spindles when incubated at the permissive temperature in the presence of HU. Previous data indicate that overexpression of *ASE1* or *CIN8* induces spindle elongation in HU-arrested cells as well. With these approaches, we demonstrate, on the basis of the following observations, that premature spindle elongation in HU-arrested yeast cells results in improper chromosome-microtubule interaction. First, elongated spindles during S-phase led to pronounced anaphase entry delay. Moreover, the delay was abolished by the inactivation of the spindle checkpoint that monitors defects in kinetochore-microtubule interaction. Finally, suppression of the premature spindle elongation by *cin8Δ* deletion alleviated the anaphase entry delay in *ask1-3* mutant cells after HU treatment.

### The Relative Timing of Centromere Duplication and Kinetochore–Microtubule Interaction in Budding Yeast

Budding yeast cells form spindles and establish the kinetochore-microtubule interaction during late S-phase [9], therefore, chromosome biorientation and spindle pole separation normally occur at about the same time. The duplication of centromeric DNA during early S-phase disrupts kinetochore structure, resulting in detached chromosomes. The recapture of these chromosomes occurs immediately after the replication of centromeric DNA, when two spindle poles have not clearly separated yet [38]. In HU-arrested cells, two spindle poles separate as a consequence of spindle formation, but DNA synthesis is blocked. Our ChIP analysis demonstrates decreased association of kinetochore protein Nnf1 and Ask1 with centromeric DNA, suggesting that the duplication of some centromeric DNA is not finished yet in HU-treated cells, even though centromeres are close to early replication origins. As a consequence, bipolar attachment cannot form on these unduplicated centromeres in HU-arrested cells. After HU is washed away, duplicated sister kinetochores allow the establishment of bipolar attachment, when spindle poles have separated already. Therefore, it is likely that HU treatment uncouples chromosome biorientation and spindle pole separation.

### The Length Control of S-Phase Spindle in Budding Yeast

Although the two spindle poles have separated in HU-arrested yeast cells, they are relatively close and S-phase spindle is shorter than that in metaphase cells. Recent work from the Bachant lab suggests that bipolar chromosome attachments provide a force to prevent spindle extension during HU arrest, as *ask1-3* and other kinetochore mutant cells exhibit elongated spindles when incubated in 37°C medium containing HU. We found that *ask1-3* cells exhibited partially elongated spindles in HU-arrested cells even when incubated at room temperature. The examination of spindle elongation kinetics reveals no noticeable difference in WT and *ask1-3* mutants in undisturbed cell cycle at 25°C. Then why do these mutants exhibit abnormal spindle elongation in the presence of HU?

Our ChIP analysis indicates decreased association of kinetochore proteins with centromeric DNA, suggesting that only a few chromosomes have assembled sister kinetochores that are competent for bipolar attachment. Consistently, the examination of Cen4-GFP and Tub1-mCherry signals in HU-arrested cells indicates the presence of many monopolar attached chromosomes. Therefore, HU treatment imposes an increased requirement for kinetochores due to a reduction in inward forces resulting from fewer bipolar kinetochore-microtubule interactions. The kinetochore defect in *ask1*-3 mutants might result in the failure to restrain spindle elongation in the presence of HU, although the spindle is normal in undisturbed cell cycle. In support of this speculation, *dam1-DDD* and *spc24-9* mutants also exhibit elongated spindles in the presence of HU [39].

### The Spindle Length and Kinetochore–Microtubule Interaction during S-Phase

One interesting observation is that *ask1-3* mutants show elongated spindles in HU-treated cells and these cells exhibit spindle checkpoint-dependent anaphase entry delay after HU is washed away. There are two possible ways to explain the observation. As Ask1 is a kinetochore protein, one possibility is that the kinetochore defects in *ask1-3* cause spindle elongation, which in turn inhibits additional kinetochore-microtubule interaction. Alternately, the defective kinetochore-microtubule interaction in HU-arrested *ask1-3* mutants contributes directly to an increase of spindle length. We favor the first model on the basis of the following observations. First, deletion of *CIN8* suppresses the premature spindle elongation in HU-treated *ask1-3* mutants as well as the anaphase entry delay after HU exposure. Since it is unlikely that *cin8Δ* suppresses the kinetochore defects in *ask1-3* mutants, a reasonable explanation is that the suppression of spindle elongation in HU-treated *ask1-3 cin8Δ* mutant cells alleviates the anaphase entry delay after HU exposure. Moreover, Ase1-induced spindle elongation in HU-arrested cells also leads to spindle checkpoint-dependent anaphase entry delay. The kinetochores should be normal in cells overexpressing *ASE1*, as no direct kinetochore function of Ase1 has been reported in budding yeast. Therefore, we believe that the short spindle structure in HU-arrested cells is crucial for the establishment of proper kinetochore-microtubule interaction in budding yeast.

What defects contribute to the anaphase entry delay in *ask1-3* mutants after HU treatment? As the delay depends on the spindle checkpoint, one possibility is the failure of chromosome attachment. If that is the case, inactivation of the spindle checkpoint in *ask1-3* mutants would result in dramatic chromosome missegregation after HU treatment. Unexpectedly, only a small portion of these cells (4%) showed missegregated chromosome IV. Our explanation is that chromosomes are bipolar attached, but the kinetochore-microtubule interaction is not strong enough to generate tension, which prevents anaphase entry by activating the tension checkpoint. Indeed, we found that deletion of the tension checkpoint gene *SGO1* partially suppressed the anaphase entry delay in *ask1-3* mutant cells after HU exposure. Together, these results support the conclusion that the presence of both unattached kinetochores and tension defects prevent anaphase entry in HU-treated *ask1-3* mutant cells.

Short S-phase spindle ensures that kinetochores are close to the two spindle poles, which may facilitate chromosome capture. In S-phase cells with elongated spindles, however, kinetochores are close to one of the spindle poles but far away from the other, making it more difficult to achieve bipolar attachment. On the other hand, even after the achievement of bipolar attachment, the unequal distance between sister kinetochores and the two spindle poles may hinder tension generation on sister kinetochores. Therefore, it becomes more difficult to achieve bipolar attachment and tension establishment when spindles are elongated prematurely in yeast cells arrested in S-phase.

## Materials and Methods

### Yeast Strains, Growth, and Media

The relevant genotypes of the yeast strains are listed in Table S1 (supporting information). All the strains listed are isogenic to Y300, a W303 derivative. They were constructed by means of standard genetic crosses. *cin8Δ* and *ase1Δ* mutants were constructed according to a PCR-based protocol [40]. To arrest yeast cells in G_1_-phase, 5 µg/ml α-factor was added into cell cultures (YPD pH 3.9) in mid-log phase (OD_600_ = 0.4) for 2–3 hr. For *CIN8* and *ASE1* overexpression, galactose was added into the medium to the final concentration of 2%.

### Protein Techniques

The preparation of protein samples was carried out as described previously [41]. Briefly, 1.5 ml of cell culture was collected and 50 µl of 20% TCA and glass beads were added to the tubes. After cells were broken with a bead beater, proteins were precipitated by centrifugation at 3000 rpm for 1 min. Equal volumes (50 µl) of 1 M Tris base and protein loading buffer were added. Protein samples were resolved by SDS-PAGE. Primary antibodies (anti-myc) were purchased from Covance (Madison, WI), and anti-Pgk1 antibody was from Molecular Probes (Eugene, OR). HRP-conjugated secondary antibody was purchased from Jackson ImmunoResearch (West Grove, PA).

### Fluorescence Microcopy

Cell cultures were collected and fixed with formaldehyde at a final concentration of 3.7% for 5 min at room temperature. The cells were then collected by centrifugation. After being washed once with PBS (pH 7.2), cells were resuspended in PBS for the microscopic examination (Zeiss Axioplan 2).

### DAPI Staining

Cells were collected and fixed with 70% ethanol at 4°C overnight. After being washed once with PBS, cells were stained with DAPI at a final concentration of 20 µg/ml for 5 min at room temperature. The cells were washed once with PBS and resuspended in PBS for fluorescence microscopy.

### Chromatin Immunoprecipitation Assay (ChIP)

Cell cultures were collected and fixed with formaldehyde at a final concentration of 3.7% for 15 min at room temperature. Glycine was added to a final concentration of 200 mM to stop crosslink reactions. After 5 min, the cells were washed twice with ice-cold TBS buffer (20 mM Tris-HCl, pH 7.4 and 150 mM NaCl). After being suspended in FA-lysis buffer (50 mM Hepes-KOH, pH 7.5, 140 mM NaCl, 1 mM EDTA, 1% TritonX-100 and 0.1% sodium deoxycholate) with protease inhibitors, the cells were homogenized with a bead beater. The resulting cell lysates were subjected to sonication, which fragments chromatins to 500–1000 bp pieces. After centrifugation, the supernatant of the cell lysates was treated with anti-myc antibody overnight at 4°C. The supernatants were further incubated with protein-A conjugated agarose beads (Santa Cruz Biotechnology), which is preincubated with BSA and ssDNA for 30 min on ice. The beads were collected and washed sequentially with FA-lysis, FA-500 (50 mM Hepes-KOH, pH 7.5, 500 mM NaCl, 1 mM EDTA, 1% TritonX-100 and 0.1% sodium deoxycholate), LiCl wash solution (10 mM Tris-HCl, pH 7.5, 250 mM LiCl, 0.5% NP-40, 1 mM EDTA, 0.5% sodium deoxycholate), and TES (10 mM Tris-HCl pH 7.5, 1 mM EDTA, 100 mM NaCl) buffers. The precipitates were eluted with elution buffer (100 mM Tris-HCl, pH 7.8, 10 mM EDTA, 1% SDS and 400 mM NaCl) at 37°C and then treated with protease K at 65°C overnight to remove proteins. DNA fragments were extracted with phenol/chloroform/isoamylalcohol and finally resuspended in TE buffer. PCR was performed according to a standard protocol. Cell lysates were diluted and used as input for PCR. Primers for *CEN1*, *CEN3*, and *ACT1* are the same as described [12]. The amount of template was adjusted to ensure that PCR was in the linear range.

## Supporting Information

Table S1 The relevant genotypes of the strains used in this study.
(0.06 MB DOC)
