# Supplementary material for: The Coordination of Centromere Replication, Spindle Formation, and Kinetochore–Microtubule Interaction in Budding Yeast
Source: PLoS Genet. 2008 Nov 21;4(11):e1000262. doi: 10.1371/journal.pgen.1000262 (PMC2577889; doi:10.1371/journal.pgen.1000262)
Supplement: Table S1 — The relevant genotypes of the strains used in this study. (0.06 MB DOC) [file pgen.1000262.s001.doc]

**Table S1. The relevant genotypes of the strains used in this study**

| **Strain** | Relevant Genotype | **Source** |
| --- | --- | --- |
| Y300 | *MAT***a** *ura3-1 his3-11, 15 leu2-3, 112 trp1-1 ade2-1 can1-100* | Lab Stock |
| Y1113 | *MAT***a** *ask1∆::LEU2* *ASK1-9myc-HIS3* | Elledge lab |
| 756-9-2 | *MAT***a** *NNF1-13myc::KanMX* | This study |
| YYW141 | *MAT***a** *promURA3::tetR::GFP::LEU2 CENIV::tetOX448::URA3 TUB1-mCherry::URA3* | This study |
| YYW161 | *MAT***a** *ask1-3* | Elledge lab |
| 777-2-2 | *MAT***a** *TUB1-GFP::URA3* | This study |
| 846-3-4 | *MAT***a** *ask1-3 TUB1-GFP::URA3* | This study |
| DDY2496 | *MAT***a** *dam1(S257D S265D S292D)::KanMX his3∆200 leu2-3,112 ura3-52* | Barnes lab |
| YYW139 | *MAT***a** *MTW1-3GFP::HIS3* *TUB1-mCherry::URA3* | This study |
| YYW140 | *MAT***a** *ask1-3* *MTW1-3GFP::HIS3* *TUB1-mCherry::URA3* | This study |
| 934-1-4 | *MAT***a** *promURA3::tetR::GFP::LEU2 CENIV::tetOX448::URA3* *NUF2-mCherry::HPHMX3* | This study |
| 934-19-1 | *MAT***a** *ask1-3 promURA3::tetR::GFP::LEU2 CENIV::tetOX448::URA3 NUF2-mCherry::HPHMX3* | This study |
| 300-1-1 | *MAT***a** *PDS1-18myc::LEU2* | This study |
| 890-2-4 | *MAT***a** *ask1-3 PDS1-18myc::LEU2* | This study |
| 894-3-4 | *MAT***a** *ask1-3 mad1Δ::HIS3 PDS1-18myc::LEU2* | This study |
| YHW305 | *MAT***a** *cin8Δ::Sphis5+* | This study |
| 971-12-3 | *MAT***a** *ask1-3 cin8Δ::Sphis5+* | This study |
| 971-7-2 | *MAT***α** *ask1-3 cin8Δ::Sphis5+* | This study |
| 683-15-3 | *MAT***a** *MTW1-3GFP::HIS3* | This study |
| 745-9-3 | *MAT***a** *ask1-3 MTW1-3GFP::HIS3* | This study |
| 974-11-1 | *MAT***a** *ask1-3 cin8Δ::Sphis5+ MTW1-3GFP::HIS3* | This study |
| 949-13-1 | *MAT***a** *cdc13-1 promURA3::tetR::GFP::LEU2 CENIV::tetOX448::URA3 NUF2-mCherry::HPHMX3* | This study |
| 2023-2-1 | *MAT***a** *TUB1-GFP::LEU2* | This study |
| 2023-1-1 | *MAT***a** *ask1-3 TUB1-GFP::LEU2* | This study |
| 2023-4-3 | *MAT***α** *cin8Δ::Sphis5+ TUB1-GFP::LEU2* | This study |
| 2023-8-1 | *MAT***a** *ask1-3 cin8Δ::Sphis5+ TUB1-GFP::LEU2* | This study |
| 2022-8-4 | *MAT***a** *sgo1Δ::KanMX promURA3::tetR::GFP::LEU2 CENIV::tetOx448::URA3* | This study |
| 2022-15-3 | *MAT***a** *ask1-3 sgo1Δ::KanMX promURA3::tetR::GFP::LEU2 CENIV::tetOx448::URA3* | This study |
